# GeFaST: An improved method for OTU assignment by generalising Swarm’s fastidious clustering approach

**DOI:** 10.1186/s12859-018-2349-1

**Published:** 2018-09-12

**Authors:** Robert Müller, Markus E. Nebel

**Affiliations:** 1 International Research Training Group “Computational Methods for the Analysis of the Diversity and Dynamics of Genomes”, Bielefeld University, Bielefeld, Germany; 2 Faculty of Technology, Bielefeld University, Bielefeld, Germany; 3 IMADA, Southern Denmark University, Odense, Denmark

**Keywords:** Sequence clustering, Operational taxonomic units, Microbial community analysis

## Abstract

**Background:**

Massive genomic data sets from high-throughput sequencing allow for new insights into complex biological systems such as microbial communities. Analyses of their diversity and structure are typically preceded by clustering millions of 16S rRNA gene sequences into OTUs. Swarm introduced a new clustering strategy which addresses important conceptual and performance issues of the popular de novo clustering approach. However, some parts of the new strategy, e.g. the fastidious option for increased clustering quality, come with their own restrictions.

**Results:**

In this paper, we present the new exact, alignment-based de novo clustering tool GeFaST, which implements a generalisation of Swarm’s fastidious clustering. Our tool extends the fastidious option to arbitrary clustering thresholds and allows to adjust its greediness. GeFaST was evaluated on mock-community and natural data and achieved higher clustering quality and performance for small to medium clustering thresholds compared to Swarm and other de novo tools. Clustering with GeFaST was between 6 and 197 times as fast as with Swarm, while the latter required up to 38% less memory for non-fastidious clustering but at least three times as much memory for fastidious clustering.

**Conclusions:**

GeFaST extends the scope of Swarm’s clustering strategy by generalising its fastidious option, thereby allowing for gains in clustering quality, and by increasing its performance (especially in the fastidious case). Our evaluations showed that GeFaST has the potential to leverage the use of the (fastidious) clustering strategy for higher thresholds and on larger data sets.

**Electronic supplementary material:**

The online version of this article (10.1186/s12859-018-2349-1) contains supplementary material, which is available to authorized users.

## Background

The advent of high-throughput sequencing (HTS) technologies revolutionised the research in the life sciences and the resulting massive genomic data sets provide the basis for new insights into the diversity and dynamics of biological systems. For example, contemporary studies of the diversity and structure of microbial communities often involve sequencing millions of 16S rRNA gene sequences due to, e.g., its ubiquitous nature [1]. In order to facilitate downstream analyses of the resulting huge amplicon data sets, the amplicons are commonly grouped into operational taxonomic units (OTUs). Over the years, diverse methods for OTU clustering have been developed, which can employ alignment-based or alignment-free [2] similarity measures and compute these exactly or approximately. In addition, methods differ in how they determine the clusters: (i) comparing sequences to a reference database and grouping those sequences which are similar to the same reference sequence (*closed-reference clustering*), (ii) clustering sequences based on their distances among each other (*de novo clustering*), and (iii) a combination of both using de novo clustering for those sequences that could not be assigned through closed-reference clustering (*open-reference clustering*).

As pointed out by Westcott and Schloss [3], all three approaches have their strengths and weaknesses, but de novo clustering has become a favourite one – especially because it does not depend on (the existence of) a reference database. However, traditional de novo methods (e.g. [4–6]) are criticised for their sensitivity to the input order of the amplicons and their dependence on an arbitrary fixed global clustering threshold [7].

### Swarm clustering

Swarm [8] has been devised as an exact, two-phased, agglomerative de novo clustering algorithm that overcomes above problems by iteratively extending a cluster using a local clustering threshold *t* and starting from the most abundant amplicons. Here, a cluster (or OTU) can be viewed as an edge-weighted, rooted, acyclic and undirected graph *G*=(*V*,*E*,*s*,*w*) where *V* is the set of vertices (amplicons), *E* is the set of edges (links between amplicons), *s* is the root (seed amplicon), and *w* is the weight function assigning to each edge the distance between the incident amplicons. Using a scoring function *δ*, Swarm considers the distance *d*_*δ*_ between two amplicons as the number of differences in an optimal alignment based on the given *δ*. Then, the set of partners of an amplicon *a* in an amplicon pool $\mathcal {A}$ respective to a distance function *d* and a threshold *t* is defined as $P_{d}(a, \mathcal {A}, t) = \{b \in \mathcal {A} \: | \: d(a, b) \leq t\}$, with Swarm using *d*=*d*_*δ*_.

**Fig. 1:**
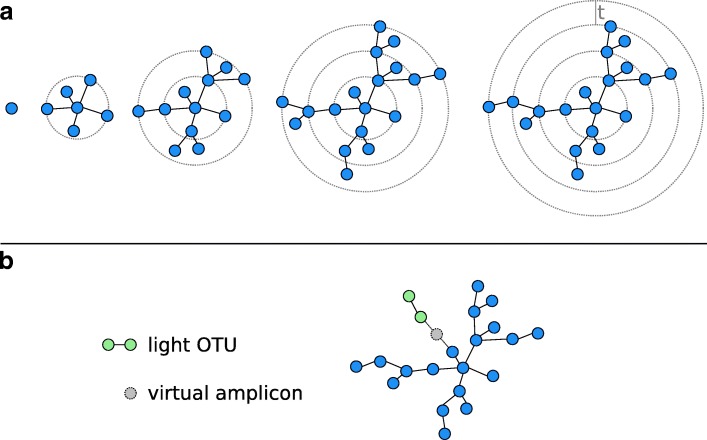
Schematic view of Swarm’s clustering strategy. **a** Starting from a seed, amplicons are added iteratively using a small local threshold *t* until the OTU reaches its natural limit when no more amplicons can be connected to it. **b** By postulating the existence of virtual linking amplicons, light OTUs are grafted onto heavy ones during the fastidious clustering step. Adapted from [8, Figure 1]

Its iterative clustering method for a pool $\mathcal {A}$ of amplicons works as follows (Fig. 1a): The most abundant amplicon in the pool is removed from it and serves as the seed *s* of a new OTU. Next, all amplicons in $P_{d_{\delta }}(s, \mathcal {A}, t)$ are transferred from $\mathcal {A}$ to the OTU (forming the first generation of subseeds). For each such subseed *s*^′^, we determine $P_{d_{\delta }}\left (s^{\prime }, \mathcal {A}, t\right)$ in order to find the second generation of subseeds. This process is iterated until no more amplicons can be added to the OTU, which is then closed. Starting with the most abundant amplicon in the remaining pool as the seed of the next OTU, the overall procedure is repeated until the pool is empty.

In order to avoid over-grouping through long chains of consecutive links between amplicons (a common problem of single-linkage clustering), Swarm also implements an optional breaking mechanism to turn different centres of abundance into separate OTUs. Originally, breaking was realised in a separate phase using a parameterised script. In brief, it examined the abundances along such amplicon chains linking centres of abundance (usually star-shaped subgraphs with an abundant amplicon in its centre, which is surrounded by less abundant amplicons) and decided on breaking or not based on the ratio between the minimum and maximum observed abundance. More current versions of Swarm use a non-parameterised breaking mechanism, which is directly included in the growth phase described above and allows only monotonically decreasing abundances along consecutive links (outwards from the seed). Partners of an amplicon are then defined as

$$\begin{array}{*{20}l} P_{d}^{\prime}(a, \mathcal{A}, t) &= \{b \in \mathcal{A} \: | \: d(a, b) \leq t \\ & \wedge a.abundance \geq b.abundance\}. \end{array} $$

Moreover, Swarm offers a so-called *fastidious* clustering option for *t*=1 from version 2.1.0 onwards in order to reduce the effect of under-grouping. To this end, Swarm distinguishes between *light* and *heavy* OTUs using a user-definable threshold *b* on their total abundance (with the sum of the abundances of the comprised amplicons being considered as the weight of an OTU). For a collection of OTUs $\mathcal {C}$ and threshold *b*, the light and heavy OTUs ($\mathcal {C}_{< b}$ and $\mathcal {C}_{\geq b}$, respectively) are defined as follows:

$$\begin{array}{*{20}l} \mathcal{C}_{< b} &= \left\{C \in \mathcal{C} \: \left| \: \underset{a \in C.V}{\sum\limits} a.abundance < b \right.\right\} \\ \mathcal{C}_{\geq b} &= \left\{C \in \mathcal{C} \: \left| \: \underset{a \in C.V}{\sum\limits} a.abundance \geq b \right.\right\} \end{array} $$

Fastidious clustering grafts light OTUs onto heavier ones by postulating the existence of a (virtual) linking amplicon (Fig. 1b). If such a virtual amplicon bridges the gap of size at most *t*_*f*_=2 (with *t*_*f*_ being the fastidious threshold) between the OTUs, then all amplicons of the light OTU (but not the virtual amplicon itself) are added to the heavy one.

In general, Swarm identifies the partners of an amplicon by iterating over the remaining amplicons in the pool and computing pairwise optimal alignments to determine the number of differences. In order to avoid a large number of unnecessary alignment computations, two amplicons have to pass a filtering step first, which compares their *k*-mer compositions to obtain an estimate of their similarity [9]. Furthermore, Swarm speeds up the alignment computations by parallelisation through SIMD instructions. For *t*=1, current versions of Swarm employ a dedicated algorithm which scales linearly with the number of amplicons. The partners of an amplicon are found by generating the microvariants of the current amplicon (i.e. all amplicons with an edit distance of 1 to it) and searching these in a hash table of the amplicons in the pool. Microvariants are also used in the fastidious clustering step, which is implemented with the help of a Bloom filter [10], a probabilistic dictionary, in which the microvariants of all amplicons of light OTUs are stored. Subsequently, the microvariants of the amplicons of heavy OTUs are cross-checked against the dictionary in order to identify the fastidious links.

### Pass-Join

As described in the previous section, determining the partners of the current subseed is a crucial step in the clustering strategy of Swarm. While the employed *k*-mer filter helps to avoid many unnecessary alignment computations, iterating over the remaining pool for each subseed is still time-consuming. Similarly, setting up the Bloom filter and cross-checking microvariants for fastidious clustering can be expensive in terms of runtime and memory consumption. Both tasks come down to identifying similar sequences, which can be efficiently accomplished by adapting the segment filter introduced by Li et al. in Pass-Join [11], a tool originally proposed for computing string similarity joins on two sets of strings using the edit distance. It follows a filter-and-verify approach to determine pairs of similar sequences efficiently, avoiding large proportions of unnecessary sequence comparisons. Li et al. also proved that their approach is both correct and complete, i.e. it finds all pairs of similar sequences and only those.

The filtering step is based on a pigeonhole principle. For a given edit-distance threshold *t*, consider two sequences *R* and *S* where *R* is divided into *t*+1 (disjoint) segments. Then, *S* has to contain a substring matching a segment of *R* if the edit distance *d*_*e*_ between *R* and *S* is at most *t*. The segments for this method are chosen using an even-partitioning scheme, limiting the maximum length difference of segments of *R* to 1.

In order to apply the pigeonhole principle efficiently, inverted indices mapping segments onto sequence identifiers are built. Hence, for each sequence length *l* and segment index (*i*∈[1:*t*+1]), the corresponding inverted index $\mathcal {I}_{l,i}$ establishes the relation between observed segments and all sequences of length *l* containing them as their *i*-th segment.

For a given set of sequences $\mathcal {S}$, we can then find (potentially) similar sequences by querying a subset of the inverted indices (chosen based on the length of the currently considered *S*) with a selection of substrings from *S*. Pass-Join finds similar sequences in a set of sequences using the pigeonhole principle and the inverted indices as described in Algorithm 1.


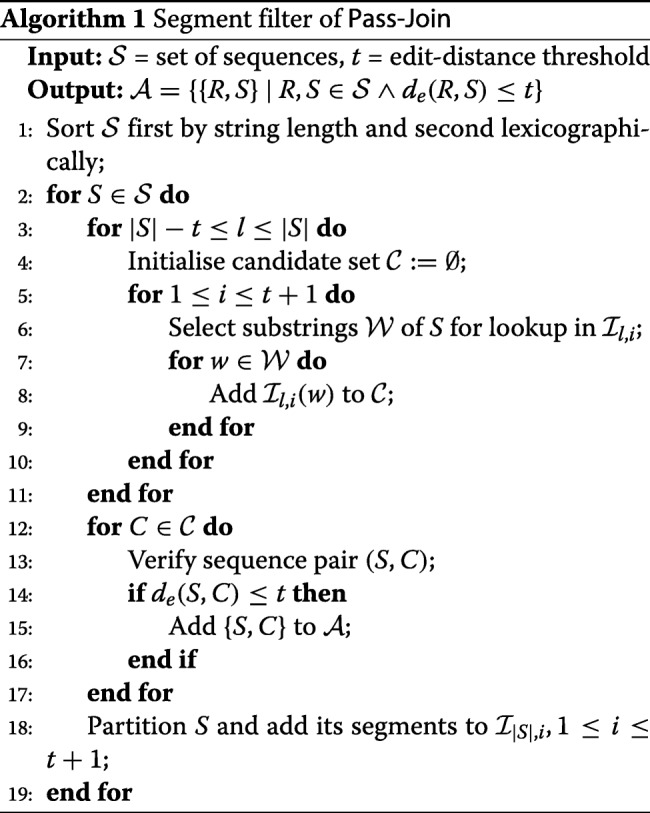


Li et al. also propose some sophisticated methods for the substring selection (Algorithm 1, line 6), reducing the number of feasible substrings to only a few per segment. Their most advanced method, *multimatch-aware substring selection*, makes use of the length and position of the segment as well as of the length difference of *S* and the indexed sequences in question and prunes the substring set by some clever considerations on where further matching substrings have to exist to satisfy the edit-distance threshold.

Furthermore, Li et al. suggest to reduce the complexity of the verification step (Algorithm 1, line 13) by computing only the bounded edit distance. They also improve on the traditional method [12] by, e.g., considering the length difference of *S* and *C* as well as adding an early-termination check. In the present study, we lift some of the restrictions of Swarm by introducing our exact, alignment-based de novo clustering tool GeFaST (**Ge**neralised **Fa**stidious **S**warming **T**ool), which in particular generalises the fastidious clustering option and makes it more broadly applicable. We assess the extended functionality in comparison with Swarm and other de novo tools by evaluating the clustering quality and performance on mock-community and natural data sets.

## Implementation

GeFaST generalises the clustering strategy of Swarm and combines it with a refined version of the segment filter introduced in Pass-Join in order to find the pairs of similar amplicons more efficiently during the computation of the OTUs. Our tool mimics the key features of Swarm and offers a similar command-line interface.

**Fig. 2:**
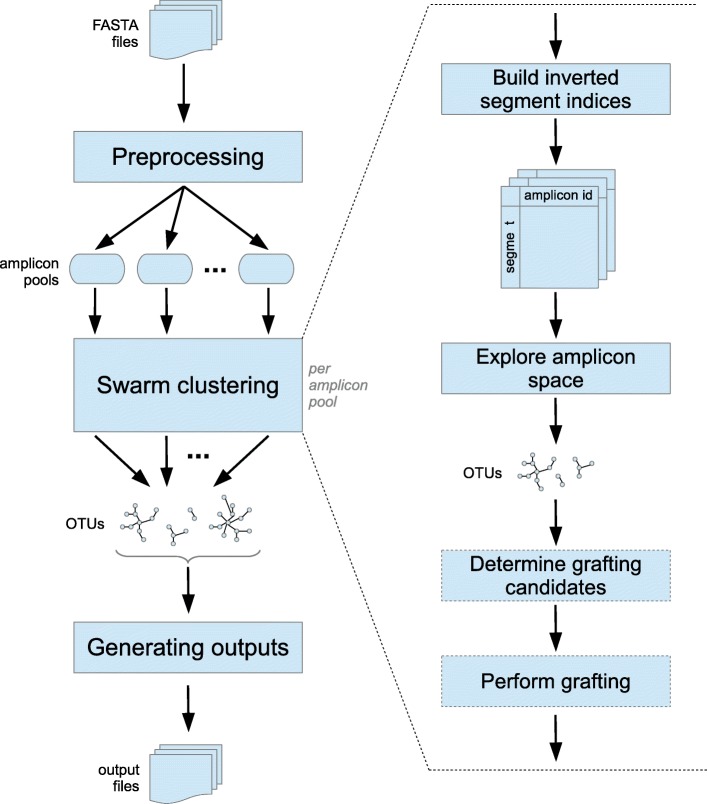
Workflow of GeFaST. The amplicons from one or more input files are preprocessed and grouped into pools. Within each pool, OTUs are formed by finding similar sequences using a segment filter. Optionally (denoted by dashed frames), OTUs can be refined by fastidious clustering. Finally, the different kinds of output are generated from the OTUs of all amplicon pools

The overall workflow of GeFaST (Fig. 2) consists of three main phases: preprocessing, swarm (or OTU) clustering and generating the outputs. The preprocessing allows to filter the input amplicons by length and alphabet. It also splits the overall set of amplicons into pools based on the clustering threshold *t* such that amplicons from different pools cannot be similar. As a result, each amplicon pool can then be handled separately in the clustering phase whose details are described below. Finally, the requested outputs are generated from the obtained OTUs, with GeFaST offering the same five output types as Swarm.

Currently, the memory consumption of our tool is in $\mathcal {O}(T)$ words where *T* is the total length of all amplicons. GeFaST’s runtime complexity is dominated by the verifications (Algorithm 1, line 13) having an overall worst-case complexity in $\mathcal {O}(N^{2} \cdot L \cdot t)$, with *N* and *L* being the number of amplicons and their maximum length, respectively.

### Implementation details.

Since GeFaST differs from the original versions of Swarm and Pass-Join’s segment filter, we subsequently describe the key aspects of our implementation.

**Segment filter.** In order to enhance the segment filter, GeFaST deviates from its original version introduced by Li et al. in some respects. First, it applies a generalised pigeonhole principle [13], dividing amplicon sequences into *t*+*k*, *k*≥1, segments of which at least *k* have to be matched. Second, GeFaST implements a bidirectional segment filter [14] adding a pipelined second filtering step in order to increase the filtering capacity. Unlike in Pass-Join, all inverted indices (per pool) are constructed at once, because the amplicons are processed in an order based on their abundance (and not their length).

**Non-fastidious clustering.** This first and mandatory clustering step explores the amplicon space in order to find the initial OTUs. For each amplicon pool, we start by building the inverted indices of the segment filter using all amplicons of this pool in order to facilitate the efficient computation of the amplicon partners. Subsequently, we determine the OTUs according to the iterative strategy described in “Swarm clustering” section. Algorithm 2 provides a pseudocode description of how the amplicon space is explored in GeFaST. The optional breaking mechanism in our tool is identical to the non-parameterised one used in newer Swarm versions. The resulting OTUs are then handed over to the fastidious clustering step or directly to the output phase.


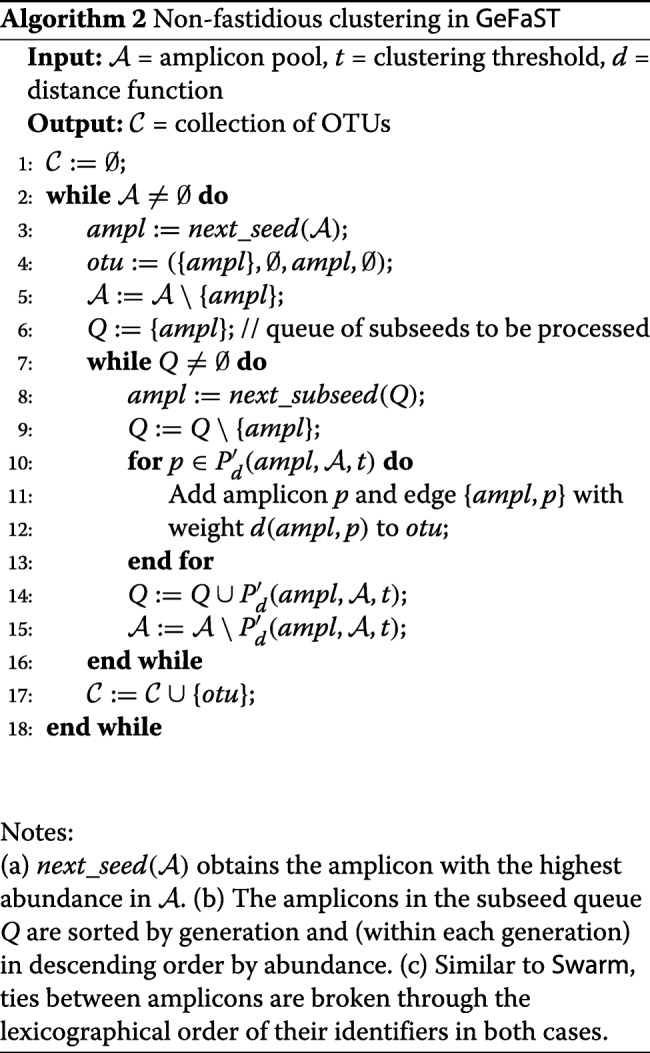


**Fastidious clustering.** The second but optional clustering step tries to refine the initial OTUs as also outlined in “Swarm clustering” section. GeFaST generalises the fastidious clustering in two ways. First, it is no longer restricted to input threshold *t*=1. This is achieved by employing a second segment filter, for which we index only the amplicons from light OTUs and search grafting partners for the ones from heavy OTUs among them. In order to preserve the idea of a virtual linking amplicon, the segment filter is used with a fastidious threshold *t*_*f*_=2∗*t* (as the default setting). Second, we capitalise on the flexibility of the segment filter by making *t*_*f*_ freely adjustable and independent of *t*. This allows for more or less conservative fastidious clustering as needed.

Subsequently, we provide a more formal description of fastidious clustering in GeFaST. A grafting link can only be established between an amplicon from a light OTU and another one from a heavy OTU. Let

$$L_{b}(\mathcal{C}) = \bigcup\limits_{otu \in \mathcal{C}_{< b}} otu.V, \:\:\:\:\:\: H_{b}(\mathcal{C}) = \bigcup\limits_{otu \in \mathcal{C}_{\geq b}} otu.V $$

be the collections of amplicons from all light and heavy OTUs, respectively. The set of potential grafting links is then defined as

$$\begin{array}{*{20}l} \mathcal{L}_{d}(\mathcal{C}, t, b) = & \{(h,l) \: | \: h \in H_{b}(\mathcal{C}) \wedge l \in L_{b}(\mathcal{C}) \\ & \wedge d(h,l) \leq t\}. \end{array} $$

For an amplicon $l \in L_{b}(\mathcal {C})$, there can be multiple potential grafting partners $h \in H_{b}(\mathcal {C})$, but only the one with the highest abundance is actually considered during the grafting process. Furthermore, a light OTU is grafted at most once, even if there are potential grafting links to several heavy OTUs. Hereinafter, we assume that $\mathcal {L}_{d}(\mathcal {C}, t, b)$ is sorted such that for all (*h*_*i*_,*l*_*i*_) and (*h*_*j*_,*l*_*j*_) with *i*<*j* the following holds

$$\begin{array}{*{20}l} h_{i}.&abundance > h_{j}.abundance \\ & \vee (h_{i}.abundance = h_{j}.abundance \\ & \wedge l_{i}.abundance > l_{j}.abundance). \end{array} $$

Finally, the valid grafting links, which are used in the fastidious clustering step (Algorithm 3), are defined as

$$\begin{array}{*{20}l} &\mathcal{V}_{d}(\mathcal{C}, t, b) = \left\{\left(h_{i}, l_{i}\right) \in \mathcal{L}_{d}(\mathcal{C}, t, b) \: | \: \right. \\ &\left.\neg\exists\left(h_{j}, l_{j}\right) \in \mathcal{L}_{d}\left(\mathcal{C}, t, b\right).\left(j < i \wedge otu\left(l_{i}\right) = otu\left(l_{j}\right)\right)\right\} \end{array} $$

where *otu*(*a*) denotes the OTU containing amplicon *a*.


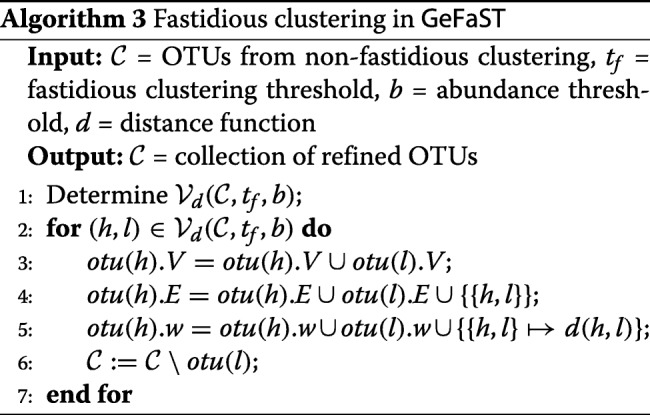


**Edit-distance mode.** The segment filter was originally developed just for the edit distance *d*_*e*_, while Swarm uses *d*_*δ*_ - based on some (user-specified) affine scoring function *δ* - as the distance between two amplicons. However, we can use the segment filter in this case (to which we will refer as *scoring-function mode*) as well, because it provides a lower bound for the number of differences in an optimal alignment. Moreover and in contrast to Swarm, the user can choose whether to run GeFaST in edit-distance or scoring-function mode.

**Verification** In order to verify whether two amplicons are similar or not, we use the length-aware verification method [11, Sec. 5.1.]. This dynamic-programming algorithm improves on a method developed by Ukkonen [12] to determine the bounded edit distance, reducing the number of diagonals to compute, and performs an early termination when it is guaranteed that the amplicons cannot be similar. In order to attain similar benefits in the scoring-function mode, we transfer the ideas of length-aware verification to Gotoh’s algorithm [15] for affine scoring functions.

## Results

In order to evaluate the performance of our tool as well as the clustering quality of the new fastidious clustering options, we conducted several comparative analyses on the following mock-community and natural data sets:

- even: The even mock-community data set from the original Swarm paper [7]. Genome isolates of the V4 region of the 16S rRNA gene from 49 bacterial and 10 archaeal species were dereplicated to 143,162 unique amplicons of average length 271.2 bp (from 1,577,469 raw reads). More information on the composition of the mock community is available in Additional file 1: Section 1.
- uneven: The uneven mock-community data set from the original Swarm paper [7]. Genome isolates of the same origin as those for even were dereplicated to 55,621 unique amplicons of average length 263.6 bp (from 637,871 raw reads). In order to obtain a more realistic community structure (including a few abundant and many rare organisms), the genome isolates were distributed according to a log-normal distribution whose parameters were fitted from a soil microbial community.
- eldermet: Natural data set obtained from the faecal microbiota of 170 human subjects as part of the ELDERMET project [16]. The 16S rRNA gene V4 region reads of all subjects were pooled and dereplicated to 4,183,843 unique amplicons of average length 250.8 bp (from 8,989,448 raw reads).

The dereplication of above data sets was performed using Swarm (v2.1.13) and, in addition, all reads that contained at least one ambiguous base (IUPAC code n resp. N) were removed. In our evaluations, we compared the de novo clustering tools GeFaST (v1.0.0), Swarm (v1.2.3 and v2.1.13), USEARCH ([4], v10.0.240_i86linux32), VSEARCH ([17], v2.7.1), CD-HIT ([6], v4.6.8), DNACLUST ([5], release 3) and Sumaclust ([18], v1.0.31). USEARCH (cluster_fast, cluster_smallmem) and VSEARCH (cluster_fast, cluster_smallmem, cluster_size) were included with different options and sorting criteria (abundance, length).

### Evaluation of clustering quality

We assessed the clustering quality using ground truths and three metrics analogous to Mahé et al. [7]: the *recall*, measuring the proportion of amplicons from the same species that are grouped in the same OTU, the *precision*, quantifying the extent to which amplicons in an OTU are also from the same species, and - summarising both - the *adjusted Rand index* [19, 20], measuring the agreement between the OTUs and the taxonomic assignment and correcting for chance.

#### Clustering mock-community data

First, we examined uneven and even with all the above tools using threshold *t* from 1 to 10 (resp. 0.99 to 0.90). Moreover, Swarm (v2.1.13) was executed with fastidious clustering for *t*=1, while GeFaST was also run with an activated fastidious option for all thresholds *t*, once per fastidious threshold *t*_*f*_∈{*t*+1,2∗*t*}. The 16S reference data set for this analysis had been hand-picked from the Greengenes database [21] by the authors of Swarm based on the list of organisms in the mock communities as pointed out by Mahé et al. (pers. comm., 2017). To ensure reproducibility, the reference data set is accessible online and a link to it is included in Additional file 1: Section 1. For both mock communities, the ground truth was established by matching the sequences against above reference data set (through VSEARCH with a minimum sequence identity of 97% and the usearch_global option) and picking the closest hit. 83.2% of the sequences in uneven and 68.2% of the ones in even matched against the reference.

**Fig. 3:**
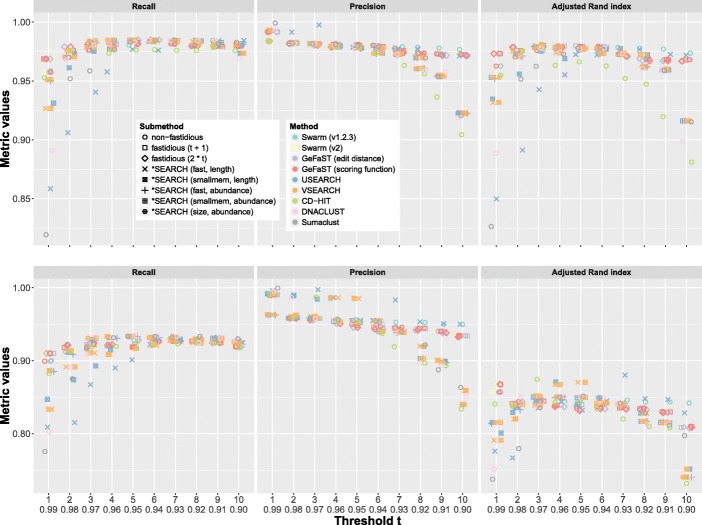
Comparison of clustering quality on uneven (*top*) and even (*bottom*) mock-community data set for ten different thresholds. Precision and recall (summarised in the adjusted Rand index) use the amplicons’ taxonomic assignments as the ground truth

The clustering quality behaved similarly on both mock-community data sets (Fig. 3). In general, the recall improved up to a threshold around *t*=6, after which it levelled of or decreased slightly. The precision declined with increasing *t* for all tools but they differed notably in the extent of this decline. Only GeFaST, Swarm and one option of USEARCH could avoid larger drops for thresholds close to 10. With some exceptions, e.g. USEARCH (cluster_fast plus length sorting) on even, the overall clustering quality (adjusted Rand index) peaked for medium to small thresholds. The overall clustering quality of many tools dropped off at one or even both ends of the threshold range (e.g. DNACLUST). In constrast, GeFaST and Swarm remained relatively stable over all examined thresholds. Hence, they achieved a higher or similar clustering quality for the majority of thresholds (especially on uneven).

Non-fastidious clustering with GeFaST and Swarm was almost identical in terms of clustering quality. Also, the differences between GeFaST’s edit-distance and scoring-function mode on both data sets were minute. However, Swarm (v.1.2.3) exceeded Swarm (v2) and GeFaST for *t*≥7 (uneven) resp. *t*≥4 (even). Due to fastidious clustering, the recall rose (decreasingly) and the precision tended to decline (increasingly) for growing *t* on both data sets. As a consequence, the adjusted Rand index decreased again for *t*≥5 (*t*≥4) when activating fastidious clustering with *t*_*f*_=*t*+1 (*t*_*f*_=2∗*t*). For all *t*≥2, we observed on both data sets that the increase (decrease) in recall (precision) due to using *t*_*f*_=2∗*t* was larger than the one due to *t*_*f*_=*t*+1 (for *t*=1, *t*_*f*_ was obviously the same in both cases).

We also tested the statistical significance of the differences in clustering quality between the evaluated tools (see Additional files 1: Section 4, 2 and 3). The results of the performed paired *t*-tests (with a significance level of 0.05) hinted at statistically significant differences between the different modes and fastidious options of GeFaST as well as between GeFaST and other tools. The magnitude of the differences compared to the metric values was, however, very small in the majority of the cases (often even below 1%).

The results of analogous analyses based on ground truths derived with a minimum sequence identity of 95 resp. 99% are shown in Additional file 1: Section 2.

#### Clustering natural data

**Fig. 4:**
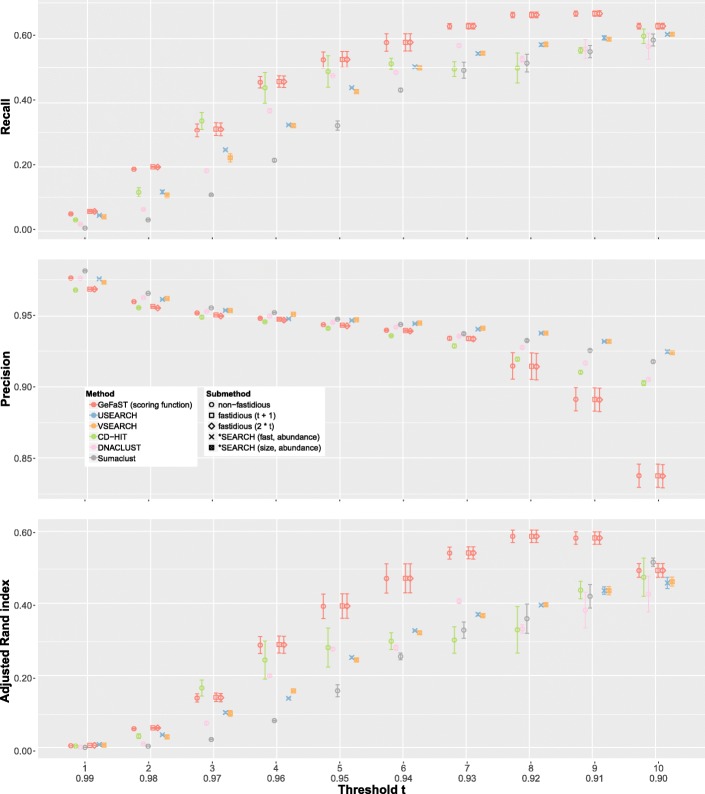
Comparison of clustering quality on the reduced eldermet data set for ten different thresholds. The average values are determined from five random subsamples (each comprising 80% of the reduced data set). The standard deviation is indicated by the error bars

Second, we performed a quality analysis on the eldermet data set at the genus level (Fig. 4) using GeFaST (as the representative of the iterative approach) as well as USEARCH, VSEARCH, CD-HIT, DNACLUST and Sumaclust (all representing the classic de novo approach). Swarm and some options of GeFaST, USEARCH and VSEARCH were not included for performance reasons or based on the results on the mock-community data. In contrast to the mock-community analyses, we had to preprocess the natural data in order to derive a feasible ground truth. In brief, we started by matching the sequences from eldermet against the SILVA database ([22], release 128) with a minimum sequence identity of 95%. Among the sequences having a match in SILVA, we kept only those that could be assigned a complete unambiguous taxonomic classification up to the genus level. The reduced eldermet data set then contained 1,315,605 unique amplicons with an average length of 244.1 bp. We conducted this analysis at the genus level because the species information in the reference databases is very incomplete and together with a minimum sequence identity of 97% less than 10% of the eldermet sequences would have passed the preprocessing. More details on the reduction steps are provided in Additional file 1: Section 3. For the actual evaluation, we generated five random subsamples of the reduced eldermet data set (each covering 80% of it). Subsequently, we computed the ground truth for each subsample and clustered each of them with the tools stated above for *t* from 1 to 10 (resp. 0.99 to 0.90).

The recall again rose with increasing threshold, but - in contrast to the previous evaluation - it started very low for all tools and achieved only a maximum recall of 0.68 through GeFaST for *t*=9. The recall of the other tools usually stayed below the one of GeFaST and hardly surpassed the level of 0.6 for *t*=10. The precision, in turn, behaved almost as for the mock communities. Starting from high values around 0.97, it decreased gradually for all tools. The precision values of the tools spread out more notably beyond *t*=7, with GeFaST showing the largest drop this time. Similar to the recall, GeFaST achieved the highest overall clustering quality with a maximum adjusted Rand index of 0.59 and usually outperformed the other tools. In contrast to the mock-community analysis, fastidious clustering did not have a notable impact on the overall clustering quality throughout this evaluation.

We again tested the statistical significance of the quality differences (see Additional files 1: Section 4 and 4). As for the mock-community data, the paired *t*-tests (with a significance level of 0.05) showed a large proportion of statistically significant differences. The magnitude of the differences compared to the metric values was, as before, very small for comparisons between different GeFaST options, but attains double-digit percentages for those involving the other tools.

### Performance evaluation

We compared the runtime and memory consumption of GeFaST (in scoring-function mode) and Swarm (v2.1.13) on eldermet in two ways. First, we used the full data set, but varied the threshold *t* from 1 to 10. Both tools were run without fastidious clustering for all thresholds. Again, the fastidious option was activated for all *t* (with *t*_*f*_ set to *t*+1 resp. 2∗*t*) for GeFaST and when possible (i.e. for *t*=1) for Swarm. Second, we examined different data set sizes while keeping threshold *t* constant. For that purpose, we randomly subsampled eldermet at various levels ranging from 5% to 100% (5% steps, three subsamples per level). Each of the 60 subsamples was then clustered with both tools for *t*∈{1,2} (the fastidious option was activated when possible as above).

In addition, we compared the performance of iterative swarm clustering and classic de novo clustering with a global threshold. To this end, we evaluated the runtime and memory consumption of GeFaST (scoring-function mode), USEARCH, VSEARCH, CD-HIT, DNACLUST and Sumaclust on the reduced eldermet data set described in the previous section. As before, threshold *t* ranged from 1 to 10 and the fastidious option of GeFaST was activated for all *t* (with *t*_*f*_ set to *t*+1 resp. 2∗*t*).

Our analyses were performed in an LXC container under Debian GNU/Linux 8.7 (jessie) on an Intel Xeon E5-2687W v4 (3.00GHz) system with 256 GB of RAM. We measured the runtime and memory consumption of a program execution via the (external) command /usr/bin/time with the resource specifiers e and M, respectively. More precisely, specifier e returns the elapsed wall clock time, while M provides the maximum resident set size.

**Fig. 5:**
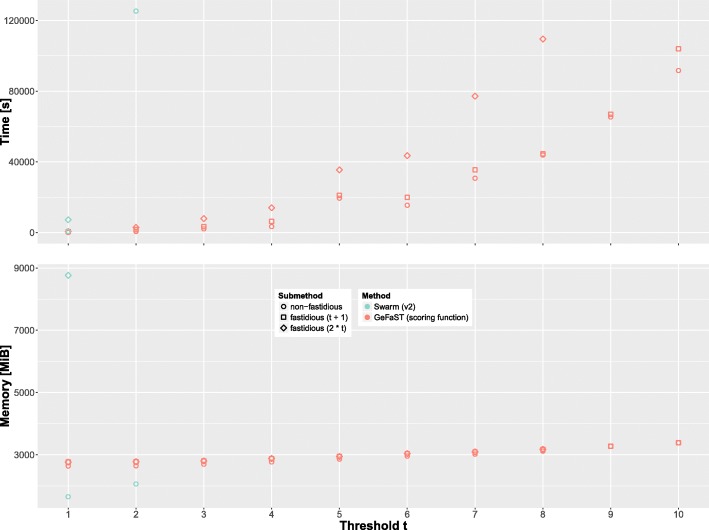
Comparison of runtime and memory consumption on eldermet for 1≤*t*≤10 with and without fastidious clustering. The runtime was capped at 36 h

**Iterative clustering for different thresholds.** The development of the runtime and memory consumption dependent on clustering threshold *t* is depicted in Fig. 5. Within the time limit of 36 h, Swarm completed the computations only for *t*≤2. In contrast, GeFaST computed all clusterings in time except for *t*=9 and *t*=10 with fastidious clustering using *t*_*f*_=2∗*t* and all these finished computations were still faster than Swarm for *t*=2. Additionally, GeFaST completed the computations for *t*≤4 (non-fastidious and fastidious with *t*_*f*_=*t*+1) resp. *t*≤2 (fastidious with *t*_*f*_=2∗*t*) in less or approximately the same time as Swarm for *t*=1 with fastidious clustering. While there was a drastic difference between the runtime of Swarm for *t*=1 and *t*=2, the runtime of GeFaST grew more gradually as *t* increased.

The memory consumption of non-fastidious clustering with GeFaST was continuously higher than the one of Swarm, while it was notably lower for fastidious clustering. Furthermore, there was a huge difference w.r.t. the amount of additional memory used for the latter. Fastidious clustering increased the memory consumption of Swarm more than fivefold, whereas GeFaST’s memory footprint grew by less than 5%.

**Fig. 6:**
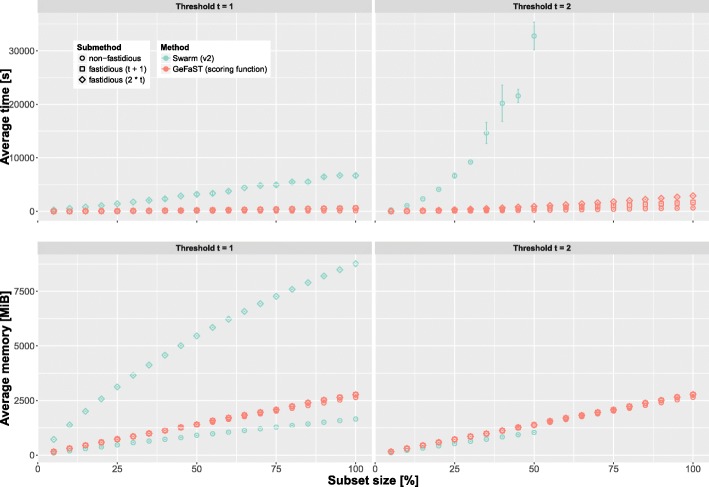
Comparison of runtime and memory consumption on differently sized subsets of eldermet for *t*=1 and *t*=2. The average values are determined from three random subsamples of the respective size, while the standard deviation is indicated by the error bars. The runtime was capped at 10 h

**Iterative clustering for different data set sizes.** Figure 6 shows how runtime and memory consumption developed with increasing data set size. GeFaST was consistently faster than Swarm for both thresholds and all fastidious options. For example, GeFaST performed non-fastidious clustering almost seven times as fast as Swarm for *t*=1. While the runtime of both tools behaved linearly in the data set size for *t*=1, they showed a non-linear behaviour for *t*=2, with Swarm displaying a much steeper incline. Hence, Swarm could only process subsets not larger than 50% of eldermet within the time limit of 10 h for *t*=2. Moreover, even fastidious clustering with GeFaST was continuously faster than non-fastidious clustering with Swarm. GeFaST also increased the runtime considerably less than Swarm across the different subset sizes. On the permutations of the full eldermet data set (i.e. the 100% subsets), for instance, the average runtime of GeFaST rose from 93 s to 585 s by activating fastidious clustering with *t*_*f*_=2, while it increased from 632 s to 6682 s for Swarm. Furthermore, the variation in the runtime on subsets of the same size tended to be stronger for Swarm.

With respect to the memory consumption, the picture is more complex. Non-fastidious clustering with Swarm was consistently more memory-efficient, but the advantage seemed to grow smaller with increasing threshold. While the average memory advantage on subsets of up to 50% of eldermet was approximately 34% for *t*=1, it was only slightly more than 26% for *t*=2. Fastidious clustering with GeFaST, in turn, required only between 22 and 32% of the memory occupied by Swarm. On top of that, the additional memory consumption due to fastidious clustering was much smaller when using GeFaST (less than 6% more memory compared to increasing fivefold or more using Swarm). In contrast to the runtime, there was no noticeable variation in the memory consumption of both tools on subsets of the same size irrespective of threshold and fastidious option.

**Fig. 7:**
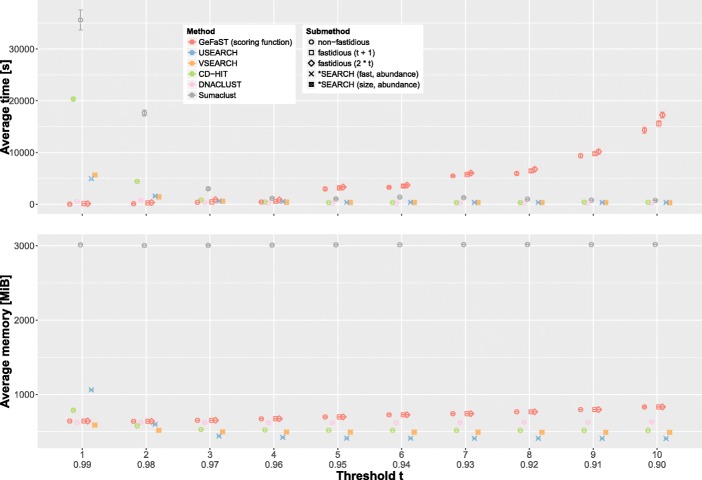
Comparison of runtime and memory consumption on the reduced eldermet data set for ten different thresholds. The average values are determined from five random subsamples (each comprising 80% of the reduced data set). The standard deviation is indicated by the error bars

**Iterative versus classic de novo clustering.** For an increasing clustering threshold *t*, GeFaST exhibited a contrary behaviour - especially w.r.t. the runtime - compared to the other tools in this evaluation (Fig. 7). While the runtime of GeFaST increased for larger thresholds, it tended to decrease for the other tools. As a consequence, GeFaST clustered the data notably faster or similarly fast for *t*≤4 but was also considerably slower for thresholds towards *t*=10. The largest gains in runtime of the non-iterative tools occurred before *t*=5, after which some of them (e.g. CD-HIT and DNACLUST) got slightly slower again. Among the non-iterative clustering approaches DNACLUST was the fastest and also the only one that showed low runtimes for thresholds down to *t*=1.

The memory consumption behaved similarly but the differences were not as distinct as for the runtime. Some non-iterative tools (e.g. USEARCH) required less memory for higher thresholds, while the memory usage of others such as DNACLUST was basically independent of *t*. The memory consumption of GeFaST was very similar to the one of the other tools for small thresholds but, in contrast to them, increased slightly for higher thresholds. However, the outlier in terms of memory usage was Sumaclust, which required more than three times as much memory than the others throughout the comparison.

## Discussion

GeFaST adds to the list of de novo clustering tools by extending the iterative approach of Swarm. Therefore, we compared GeFaST to Swarm and other current de novo tools for a range of clustering thresholds and on different taxonomy levels and kinds of data. Our analyses showed that there are notable differences between the classic approach using a global threshold and the iterative one of Swarm and GeFaST.

We evaluated the clustering quality on natural and mock-community data, with the latter being acknowledged as a trade-off between simulated and natural data by being of biological origin but also of known composition [23]. While the two mock-community data sets span a number of phyla, these often comprise more than one class and even several species per class to make the data more representative and to not rule out effects such as single-linkage chaining from the outset. On the mock-community data, GeFaST showed a smaller dependence on the clustering threshold and was often similarly good or even slightly better than the classic de novo tools. On the natural data, in contrast, the threshold had a large impact on all tools, but the clustering quality was notably higher for GeFaST. Moreover, the edit-distance and scoring-function mode (using the default parameters borrowed from Swarm) did only differ slightly, hinting at the possibility to use the potentially faster edit-distance mode without impairing the quality.

In our analyses, fastidious clustering improved the quality only on the mock-community data sets. The reasons for the lack of effect on natural data require further analysis but potential factors are a relatively low number of light OTUs and the restriction to the genus level. Based on our evaluation, benefits in clustering quality from fastidious clustering might be expected for thresholds up to 5. Beyond that the clustering became too greedy and aggregated supposedly different species, thus impairing the clustering quality. Many of the differences were found to be statistically significant but their relative size were often small as well. Hence, their practical significance is yet to be examined through, e.g., more biologically motivated metrics such as diversity measures or heritability [24].

In order to explore the limits of GeFaST, we also repeated the mock-community analysis for ground truths based on different sequence similarities. The effect of changing the ground truth was similar for all tools in our evaluation, suggesting that GeFaST is equally well-suited for OTU analyses at different levels of granularity.

Our evaluations also showed large differences between the tools in terms of their performance. Most of the classic de novo tools tended to need less time and memory for larger clustering thresholds, most likely due to the decreasing number of clusters they had to build and maintain. On the contrary, GeFaST’s runtime and, to a lesser extent, its memory consumption increased with a growing threshold. The employed segment filter was a major factor in these increases. On the one hand, the number of substrings per sequence to check during the filtering grows polynomially in the threshold. On the other hand, higher thresholds increase the number of inverted indices to be held in memory.

On top of that, there were also distinct differences between GeFaST and Swarm. As described above, increasing threshold *t* led to a relatively gradual growth in runtime for GeFaST, whereas there was a much larger change between *t*=1 and *t*≠1 for Swarm, which applies a dedicated algorithm in the former case. Compared to Swarm, the runtime of GeFaST benefits from the efficient determination of potential amplicon partners due to the segment filter and their fast verification through bounded computations with early termination. Furthermore, the effect of fastidious clustering on runtime and memory consumption was largely different between the two tools. This asymmetry stems from the use of a more memory-intensive Bloom filter and the lengthy cross-checking of microvariants in Swarm compared to another segment filter in GeFaST in order to facilitate the fastidious clustering step.

Future work is going to address GeFaST’s runtime and memory consumption as well as the achievable clustering quality. On the one hand, we will work on the performance of the segment filter for higher thresholds and explore the benefits of parallelising (parts of) GeFaST’s workflow. On the other hand, we plan to introduce memory-saving succinct data structures [25] for the key data structures of GeFaST in order to investigate their applicability to sequence clustering in terms of runtime.

With respect to the clustering quality, we will examine the effects of over- and under-grouping more closely. This will involve the exploration of alternative breaking mechanisms and the analysis of how strongly fastidious clustering affects clusters obtained by them, e.g. the one used in older versions of Swarm which produced clusters of higher quality for high thresholds during our analyses. Further evaluations of fastidious clustering will also address the effect of its parameters, i.e. the fastidious clustering threshold *t*_*f*_ and the abundance boundary *b* between light and heavy OTUs. The subsequent evaluations will include further comparisons with Swarm and other tools on mock and natural data sets (down to the species level, if possible) as well as the use of a more extensive set of metrics. As pointed out by Westcott and Schloss [3], this is sensible in order to obtain a more objective quality assessment since there is a wide range of approaches all having their assets and drawbacks.

Moreover, we will continue to investigate the characteristics and limits of GeFaST, e.g. whether there is a relation between the clustering threshold and the expected amplicon length in terms of the clustering quality or whether there is a minimum sequence length for the iterative approach to work properly.

## Conclusions

We introduced GeFaST, an exact, alignment-based de novo clustering tool which generalises the fastidious clustering approach of Swarm to arbitrary thresholds in order to reduce under-grouping in a broader range of settings. Comparisons with Swarm and other current de novo clustering tools on mock-community and natural data showed a competitive or even better clustering quality with GeFaST in a variety of settings. Some results also indicated at improvements due to fastidious clustering for small and medium thresholds up to 5 that might be beneficial to downstream analyses. In addition, our tool outperformed Swarm in terms of runtime throughout our analyses and was also faster than the other tools for thresholds up to 4. Depending on the clustering threshold and the fastidious option, GeFaST was between 6 and 197 times as fast as Swarm. However, Swarm used up to 38% less memory for non-fastidious clustering, but required at least three times as much memory as GeFaST for fastidious clustering. Furthermore, our tool scaled better with increasing data set size (especially for *t*>1) at the cost of a moderately increased memory footprint. It could also complete computations for higher thresholds and / or with fastidious clustering faster than Swarm with less demanding parameters.

## Availability and requirements

**Project name:**GeFaST**Project home page:**https://github.com/romueller/gefast**Operating system(s):** Linux**Programming language:** C++11 (developed with GCC 4.9.2)**Other requirements:** make**License:** GNU Affero General Public License v3.0**Any restrictions to use by non-academics:** None

## Additional file

Additional file 1 Supplement containing information on the mock-community data, the analyses and additional results. (PDF 336 KB)

Additional file 2 Tabular data showing results on the statistical significance of differences in clustering quality on the uneven data set. (CSV 220 KB)

Additional file 3 Tabular data showing results on the statistical significance of differences in clustering quality on the even data set. (CSV 219 KB)

Additional file 4 Tabular data showing results on the statistical significance of differences in clustering quality on the eldermet data set. (CSV 86 KB)

## Abbreviations

bp: Base pair
GeFaST: Generalised fastidious swarming tool
HTS: High-throughput sequencing
IUPAC: International union of pure and applied chemistry
OTU: Operational taxonomic unit
rRNA: Ribosomal ribonucleic acid
SIMD: Single instruction, multiple data
