# Supplementary material for: GeFaST: An improved method for OTU assignment by generalising Swarm’s fastidious clustering approach
Source: BMC Bioinformatics. 2018 Sep 12;19:321. doi: 10.1186/s12859-018-2349-1 (PMC6134716; doi:10.1186/s12859-018-2349-1)
Supplement: Supplementary file 1 — Supplement containing information on the mock-community data, the analyses and additional results. (PDF 336 KB) [file 12859_2018_2349_MOESM1_ESM.pdf]

GeFaST: An improved method for OTU assignment by generalising Swarm's fastidious clustering
Robert Müller and Markus E. Nebel

Additional file 1: Supplementary information on data and analyses

# 1 Information on mock-community data

The mock-community data sets `uneven` and `even` have been borrowed from the clustering analysis of the original Swarm paper [1]. They comprise genome isolates from 49 bacterial and 10 archaeal strains. Mahé et al. collated two strains of *Methanococcus maripaludis* and two strains of *Shewanella baltica* because they did not use taxonomic classifications beyond the species level. The 47 bacterial species belong to 17 phyla and 22 classes, while the 10 archaeal species cover three phyla and five classes. More details on the biological composition are provided in Supplement Table 1. Information on the sequencing workflow are available in the original Swarm paper [1].

The ground truth for this analysis was established by matching the sequences against a 16S reference data set. This data set was hand-picked from the GreenGenes database [2] based on the list of organisms in the mock community (Mahé et al., personal communication). Using a minimum sequence identity of, e.g., 97 %, the matching was performed through VSEARCH ([3], v2.7.1) with the `usearch_global` option and picking the closest hit.

The mock-community data sets (`even`, `uneven`) and the `references` are available online.

# 2 Evaluation on mock-community data

In order to evaluate the clustering quality of GeFaST, we first performed an evaluation very similar to the one presented in the original Swarm paper [1]. However, we included some additional and newer versions of the compared tools in our evaluation.
As described in the main article, the clustering quality was assessed via a comparison of the OTUs obtained from the clustering tools with a ground truth. This ground truth was determined as described in the previous section. The results shown in the main article are based on the common 97 % threshold targeting a distinction at the species level. The following subsections present the results of the evaluation with different similarity thresholds for more and less fine-grained OTU calling.

## 2.1 95 % ground truth

Repetition of the mock-community analysis with a ground truth determined using a minimum sequence identity of 95 % (Supplement Figure 1). 87.5 % of the sequences in `uneven` and 74.2 % of the ones in `even` matched against the reference.

## 2.2 99 % ground truth

Repetition of the mock-community analysis with a ground truth determined using a minimum sequence identity of 99 % (Supplement Figure 2). 53.0 % of the sequences in `uneven` and 36.6 % of the ones in `even` matched against the reference.

## 2.3 Subsampling

Similarly to `eldermet`, we also performed a clustering-quality analysis on random subsamples of `uneven` (Supplement Figure 3) and `even` (Supplement Figure 4). Again, we subsampled each of the two data sets five times at a level of 80 % and clustered each subsample with all tools for the different thresholds. The thresholds and metrics were the same as for `eldermet`. In contrast to the `eldermet` analysis, we did not need to reduce the data set and determined the ground truths using a 97 % minimum sequence identity on the mock-community reference data set.

| | Phylum | Class | Species / genome name |
|---|---|---|---|
| Bacteria | Acidobacteria | Acidobacteriae | Acidobacterium capsulatum ATCC 51196 |
| | Actinobacteria | Actinobacteria | Salinispora arenicola CNS-205 |
| | | | Salinispora tropica CNB-440 |
| | Aquificae | Aquificae | Hydrogenobaculum sp. Y04AAS1 |
| | | | Persephonella marina EX-H1 |
| | | | Sulfurihydrogenibium sp. YO3AOP1 |
| | | | Sulfurihydrogenibium yellowstonense SS-5 |
| | Bacteroidetes | Bacteroidia | Bacteroides thetaiotaomicron VPI-5482 |
| | | | Bacteroides vulgatus ATCC 8482 |
| | | | Porphyromonas gingivalis ATCC 33277 |
| | Chlorobi | Chlorobia | Chlorobaculum tepidum TLS |
| | | | Chlorobium limicola DSM 245 |
| | | | Chlorobium phaeobacteroides DSM 266 |
| | | | Chlorobium phaeovibrioides DSM 265 |
| | | | Pelodictyon phaeoclathratiforme BU-1 |
| | Chloroflexi | Chloroflexi | Chloroflexus aurantiacus J-10-fi |
| | | | Herpetosiphon aurantiacus ATCC 23779 |
| | Cyanobacteria | unclassified | Nostoc sp. PCC 7120 |
| | Dictyoglomi | Dictyoglomia | Dictyoglomus turgidum DSM 6724 |
| | Firmicutes | Bacilli | Enterococcus faecalis V583 |
| | | Clostridia | Anaerocellum thermophilum Z-1320, DSM 6725 |
| | | | Caldicellulosiruptor saccharolyticus DSM 8903 |
| | | | Clostridium thermocellum ATCC 27405 |
| | | | Thermoanaerobacter pseudethanolicus ATCC 33223 |
| | Fusobacteria | Fusobacteria | Fusobacterium nucleatum ATCC 25586 |
| | Gemmatimonadetes | Gemmatimonadetes | Gemmatimonas aurantiaca T-27T |
| | Planctomycetes | Planctomycetacia | Rhodopirellula baltica SH 1 |
| | Proteobacteria | Alphaproteobacteria | Rhodospirillum rubrum ATCC 11170 |
| | | | Ruegeria pomeroyi DSS-3 |
| | | | Sulfitobacter sp. EE-36 |
| | | | Sulfitobacter sp. NAS-14.1 |
| | | | Zymomonas mobilis ZM4 |
| | | Betaproteobacteria | Bordetella bronchiseptica RB50 |
| | | | Burkholderia xenovorans LB400 |
| | | | Leptothrix cholodnii SP-6 |
| | | | Nitrosomonas europaea ATCC 19718 |
| | | Gammaproteobacteria | Shewanella baltica OS185 |
| | | | Shewanella baltica OS223 |
| | | Deltaproteobacteria | Desulfovibrio desulfuricans ATCC 27774 |
| | | | Desulfovibrio piger ATCC 29098 |
| | Spirochaetes | Spirochaetes | Treponema denticola ATCC 35405 |
| | Thermi | Deinococci | Deinococcus radiodurans R1 |
| | | Thermi | Thermus thermophilus HB8 |
| | Thermotogae | Thermotogae | Thermotoga neapolitana DSM 4359 |
| | | | Thermotoga petrophila RKU-1 |
| | | | Thermotoga sp. RQ2 |
| | Verrucomicrobia | Verrucomicrobiae | Akkermansia muciniphila ATCC BAA-835 |
| Archaea | Crenarchaeota | Thermoprotei | Ignicoccus hospitalis KIN4/I |
| | | | Pyrobaculum aerophilum IM2 |
| | | | Pyrobaculum calidifontis JCM 11548 |
| | | | Sulfolobus tokodaii 7(S311) |
| | Euryarchaeota | Archaeoglobi | Archaeoglobus fulgidus DSM 4304 |
| | | Methanococci | Methanocaldococcus jannaschii DSM 2661 |
| | | | Methanococcus maripaludis C5 |
| | | | Methanococcus maripaludis S2 |
| | | Thermococci | Pyrococcus horikoshii OT3 |
| | Nanoarchaeota | Nanoarchaea | Nanoarchaeum equitans Kin4-M |

Supplement Table 1: Biological composition of the mock communities. Adapted from [1, Suppl. Tab. 1].

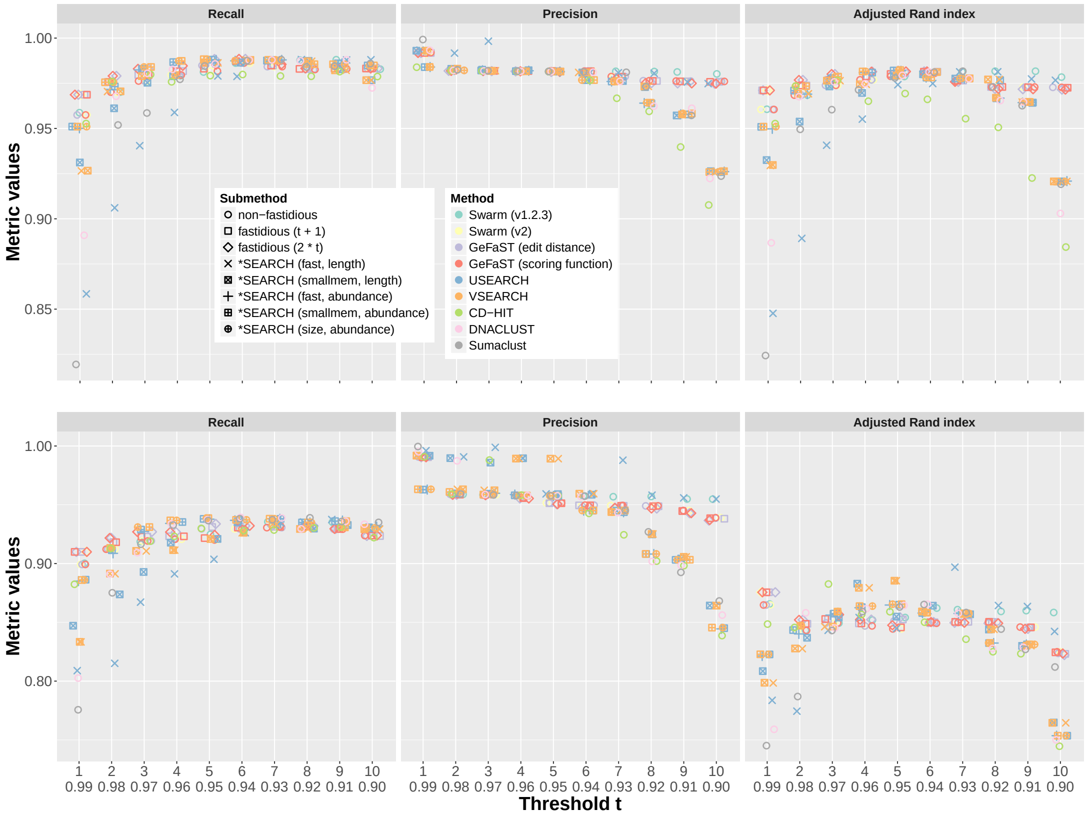


Supplement Figure 1: Comparison of clustering quality on `uneven` (*top*) resp. `even` (*bottom*) mock-community data set for 10 different thresholds using a 95 % ground truth.

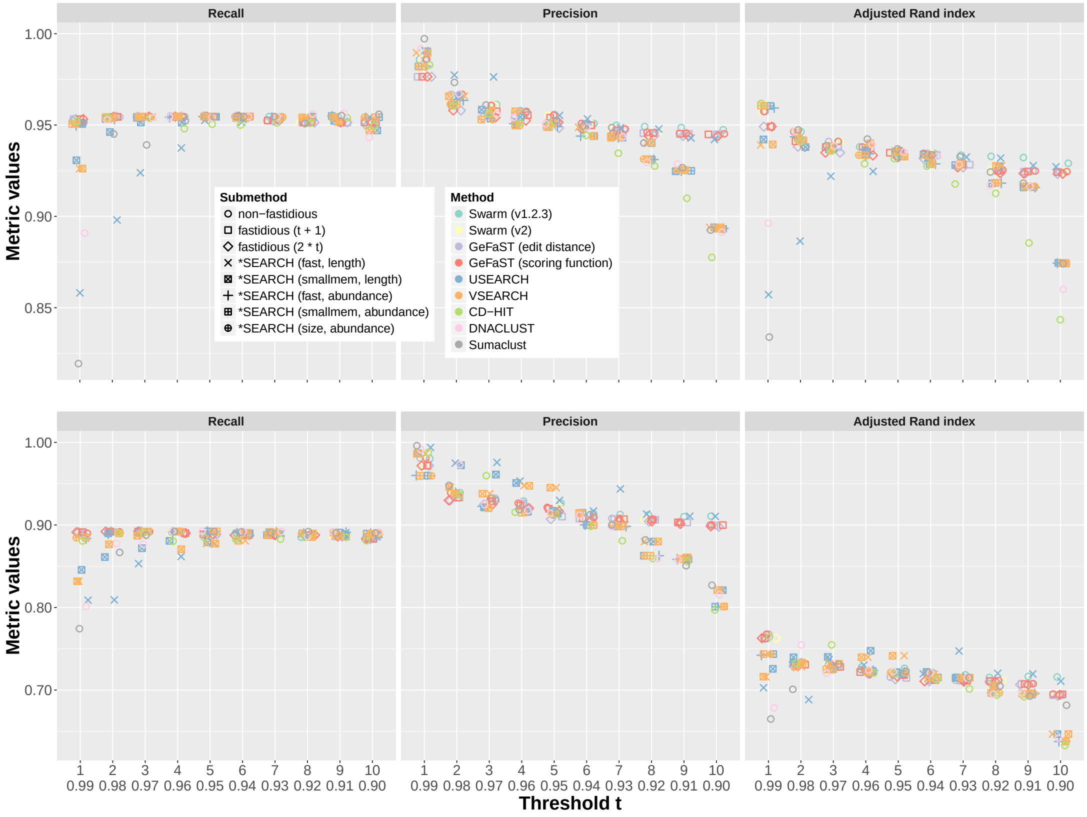


Supplement Figure 2: Comparison of clustering quality on `uneven` (*top*) resp. `even` (*bottom*) mock-community data set for 10 different thresholds using a 99 % ground truth.

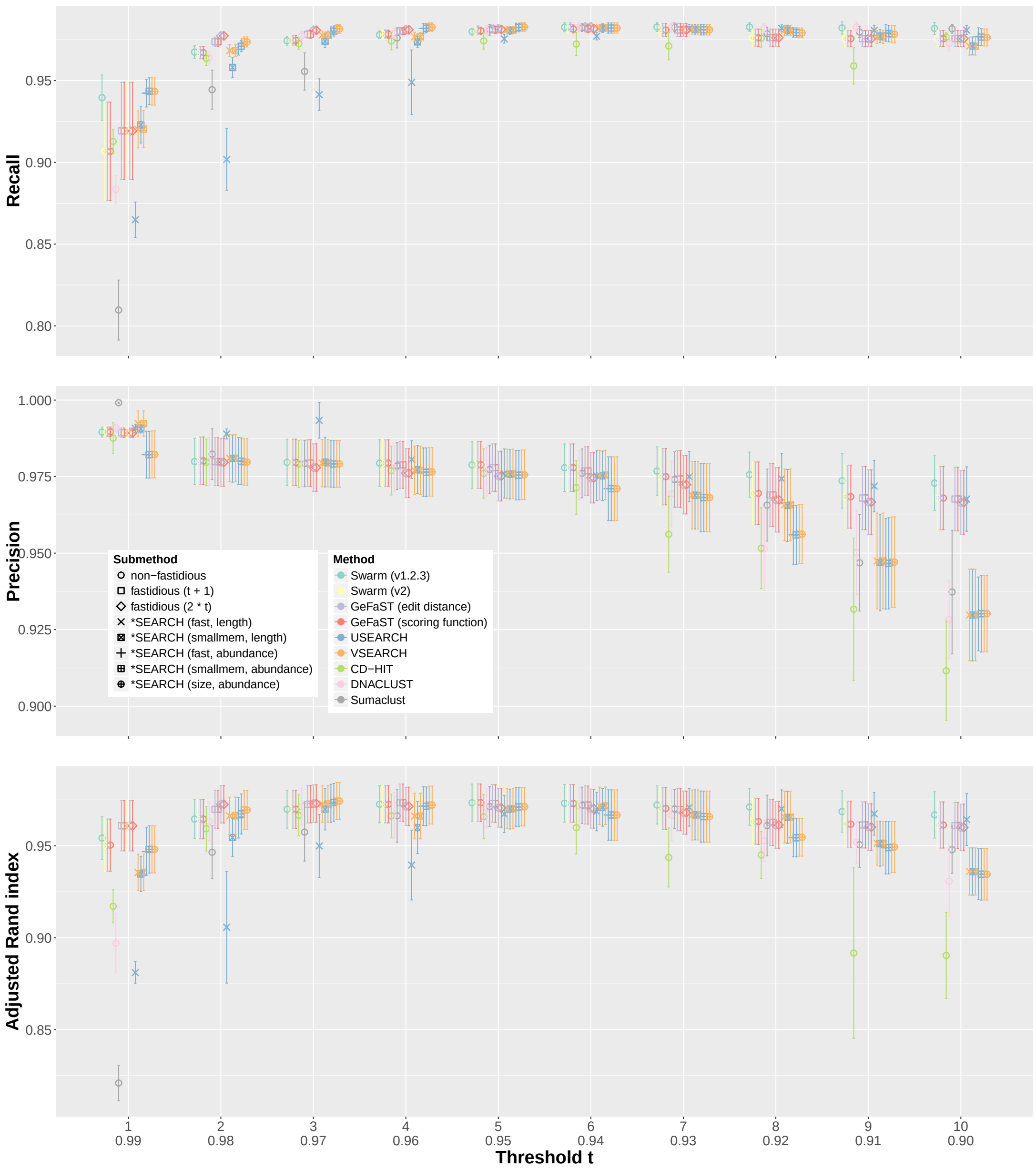


Supplement Figure 3: Comparison of clustering quality on the `uneven` data set for 10 different thresholds. The average values are determined from five random subsamples (each comprising 80 % of the data set). The standard deviation is indicated by the error bars.

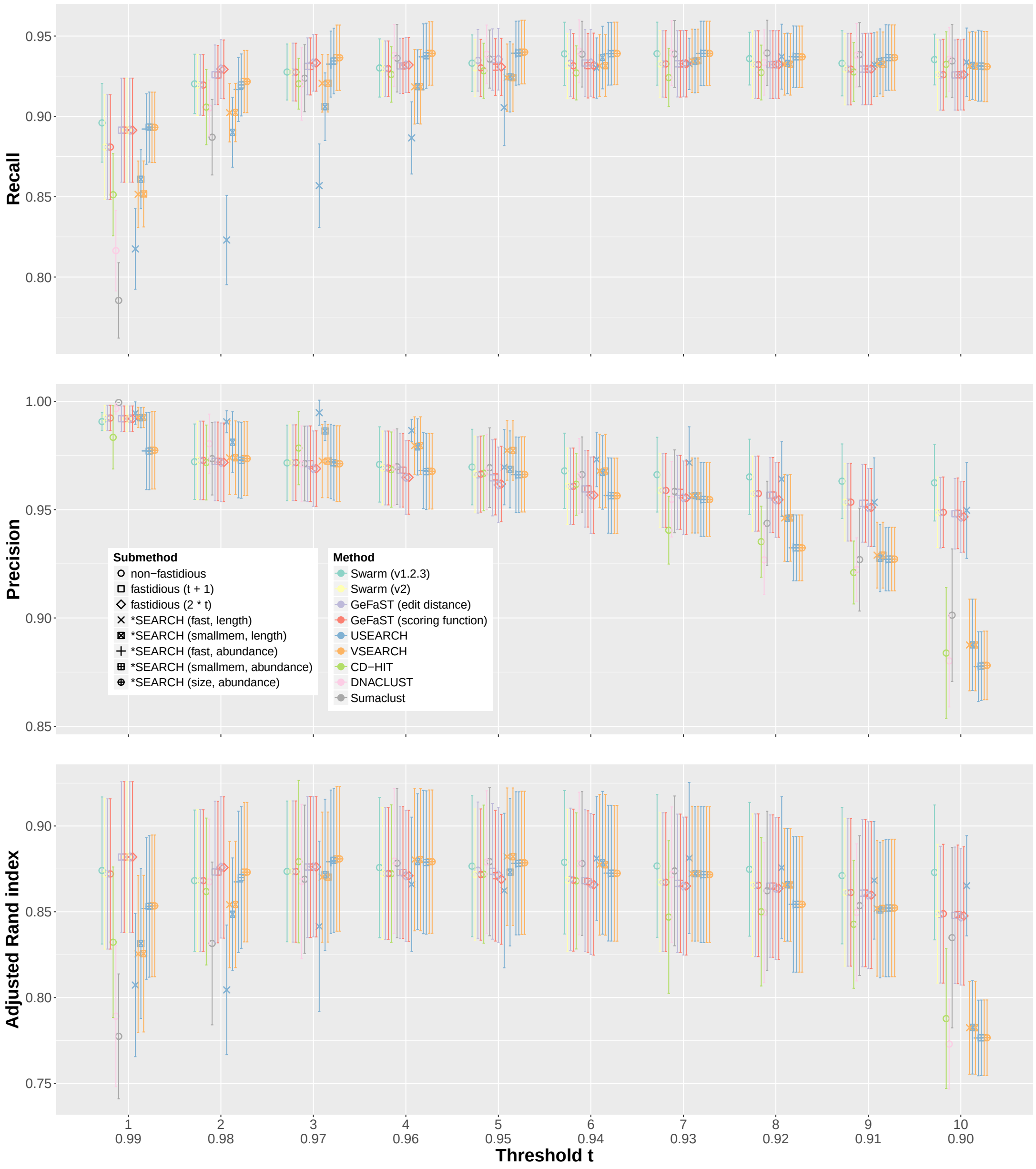


Supplement Figure 4: Comparison of clustering quality on the `even` data set for 10 different thresholds. The average values are determined from five random subsamples (each comprising 80 % of the data set). The standard deviation is indicated by the error bars.

# 3 Evaluation on natural data

In order to complement the analysis on mock-community data, we also performed an evaluation on the natural `eldermet` data set. Since establishing a ground truth on natural data is harder, the analysis was preceded by a preprocessing of the data set. While de novo clustering assigns all sequences to clusters, closed-reference clustering discards those sequences that cannot be assigned to a reference. Hence, a ground truth resulting from closed-reference clustering might cover only a small proportion of the sequences in the de novo clusters. As this can heavily skew the clustering metrics, we applied the following steps to address this issue:

1. Match the dereplicated `eldermet` data set against the 97 % representative set of the SILVA database ([4], release 128).
2. Replace the identifiers of SILVA representatives in the resulting assignment with their actual taxonomic information.
3. Remove the species-level information in the taxonomic assignment (if existent).
4. Discard entries where genus information is missing or ambiguous.
5. Reduce `eldermet` to those sequences remaining in taxonomic assignment.

The closed-reference clustering of step 1 was conducted with VSEARCH (v2.7.1) and a minimum sequence identity of 95 %. The reduced `eldermet` data set and taxonomic assignment were the inputs for the subsequent clustering-quality evaluation.

# 4 Significance of clustering-quality results

We assessed the significance of the differences in clustering quality between the different tools. To this end, we used the results of the mock-community evaluations in Section 2.3 and of the quality analysis on `eldermet` from the main article.

Here, we present an evaluation of the statistical significance of the differences between the tested tools and GeFaST (in scoring-function mode) through paired two-sided $t$-tests with a significance level of 0.05. Two methods (with certain submethods, if applicable) were compared over all examined thresholds for a set of subsamples. One $t$-test used the measurements of two methods for a specific combination of data set, metric and threshold. When only Swarm and GeFaST were involved, we used the same threshold for both methods. In a comparison between a method using a global threshold (e.g. VSEARCH) and GeFaST, we used a given local clustering threshold $t$ for GeFaST and $t' = 1 - t/100$ as the global threshold for the other method. The statistical significance is depicted in one table per data set as shown in the example below:

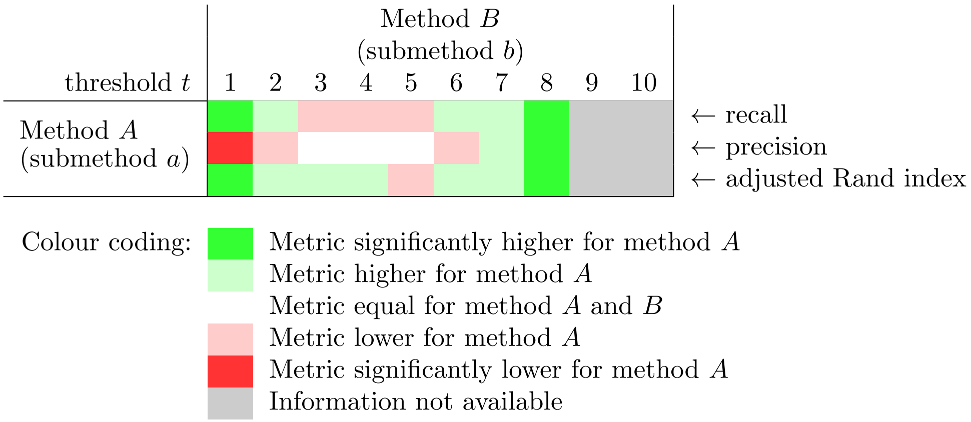


Supplement Table 2 (`uneven`) and Supplement Table 3 (`even`) show the results for the mock-community data, while Supplement Table 4 covers `eldermet`. In these tables, we abbreviated scoring function and edit distance as s.f. and e.d., respectively. The complete information underlying these tables are available in CSV format in Additional file 2 to 4. For each comparison, they state the mean and standard deviation of the differences in the respective metric as well as the $p$-value. In addition, the size of the mean difference was assessed by comparing it to the standard deviation of the differences (*power1*) and the mean value of the metric (*power2*).

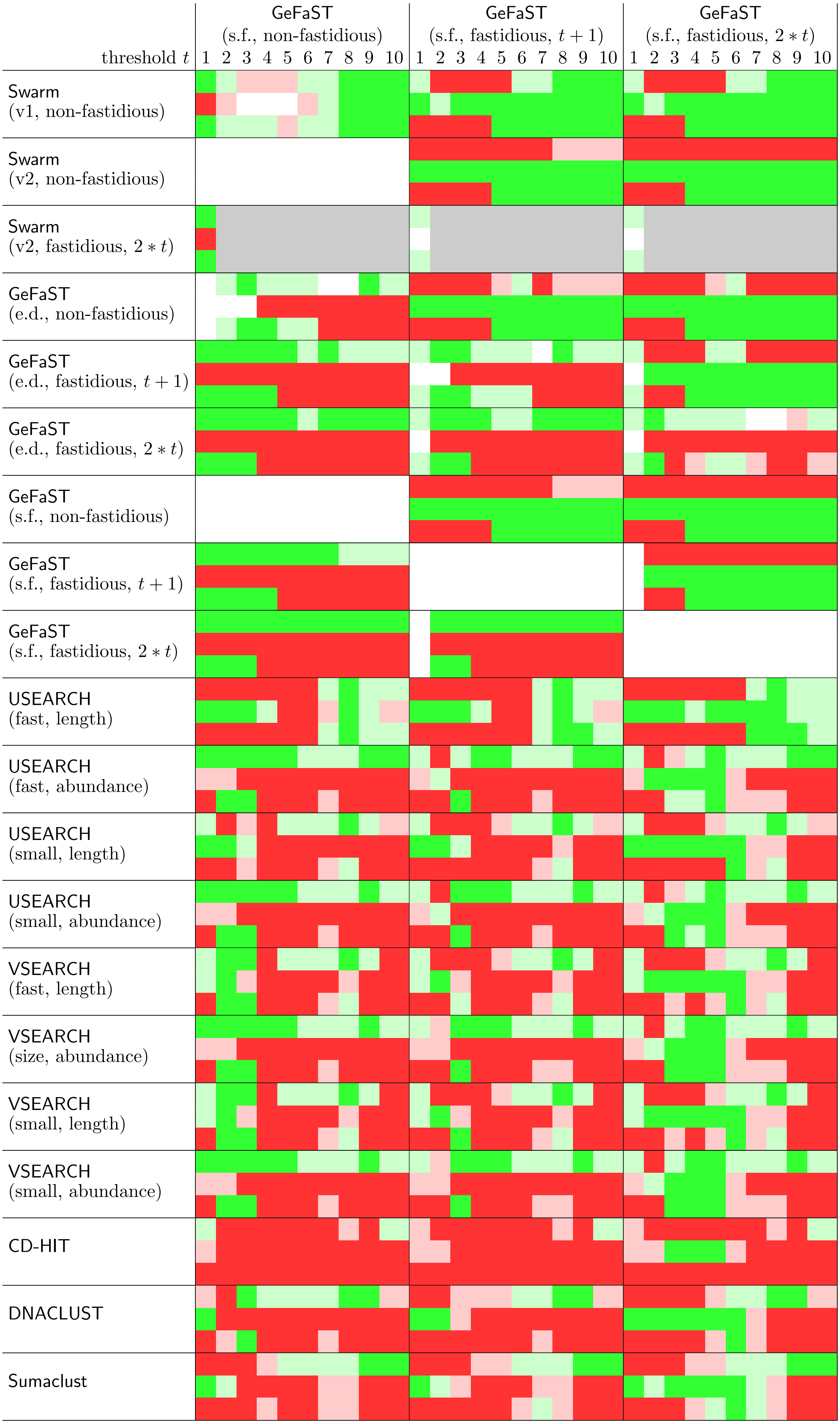


Supplement Table 2: Statistical significance of differences in clustering quality on `uneven`.

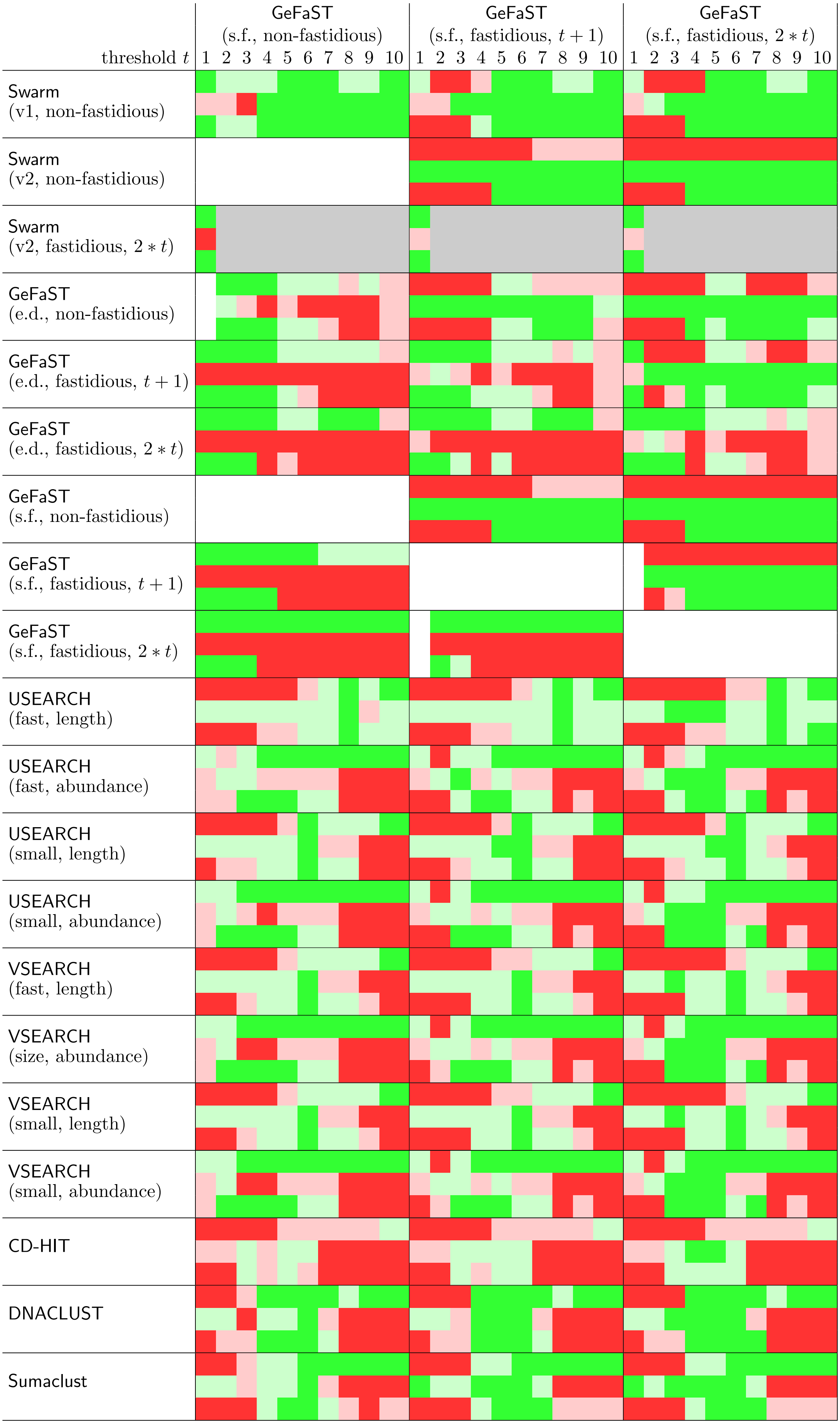


Supplement Table 3: Statistical significance of differences in clustering quality on `even`.

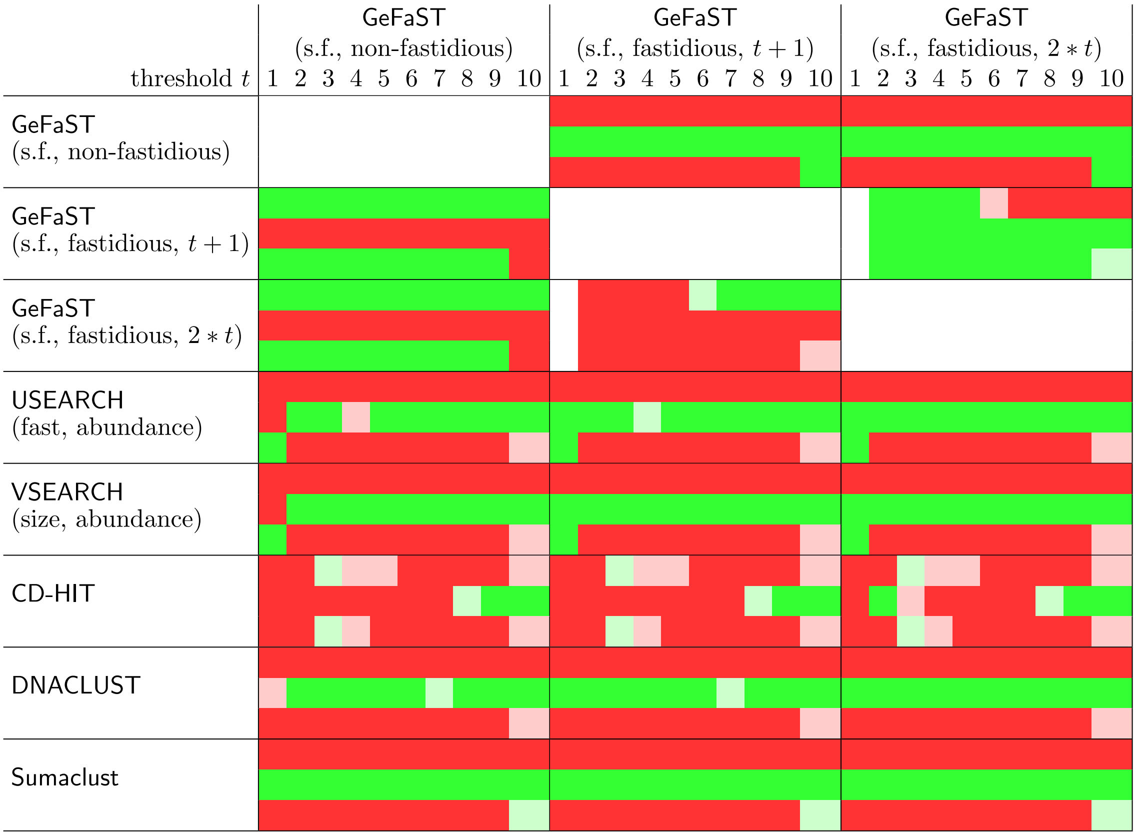


Supplement Table 4: Statistical significance of differences in clustering quality on `eldermet`.

# References


[1] Mahé, F., Rognes, T., Quince, C., de Vargas, C., Dunthorn, M.: Swarm: robust and fast clustering method for amplicon-based studies. PeerJ **2**, 593 (2014). doi:10.7717/peerj.593

[2] DeSantis, T.Z., Hugenholtz, P., Larsen, N., Rojas, M., Brodie, E.L., Keller, K., Huber, T., Dalevi, D., Hu, P., Andersen, G.L.: Greengenes, a Chimera-Checked 16S rRNA Gene Database and Workbench Compatible with ARB. Applied and Environmental Microbiology **72**(7), 5069–5072 (2006). doi:10.1128/AEM.03006-05

[3] Rognes, T., Flouri, T., Nichols, B., Quince, C., Mahé, F.: VSEARCH: a versatile open source tool for metagenomics. PeerJ **4**, 2584 (2016). doi:10.7717/peerj.2584

[4] Quast, C., Pruesse, E., Yilmaz, P., Gerken, J., Schweer, T., Yarza, P., Peplies, J., Glöckner, F.O.: The SILVA ribosomal RNA gene database project: improved data processing and web-based tools. Nucleic Acids Research **41**(D1), 590–596 (2013). doi:10.1093/nar/gks1219
